# Design and manufacturing of roller bearing polymeric cages and development of a theoretical model for predicting the roller push-out force

**DOI:** 10.1038/s41598-022-04959-9

**Published:** 2022-01-19

**Authors:** Alireza Zarei, Saeed Farahani, Sai Aditya Pradeep, John Driscoll, Rob Lukasiewicz, Srikanth Pilla

**Affiliations:** 1 Department of Automotive Engineering, Clemson University, Greenville, SC USA; 2 Clemson Composites Center, Clemson University, Greenville, SC USA; 3 JTEKT North America Corporation, Greenville, SC USA; 4 Department of Mechanical Engineering, Clemson University, Clemson, SC USA; 5 Department of Materials Science and Engineering, Clemson University, Clemson, SC USA

**Keywords:** Engineering, Mathematics and computing

## Abstract

In the present work, polymeric cages with 18 different pocket geometries are developed to investigate the effects of geometrical parameters and material properties on the amount of roller push-out force. An experimental setup including a specialized injection molding tool is designed and fabricated and three sets of polymeric cages are manufactured using the selected materials (PA46, PA66, PPA). Force measurements are carried out five times on each pocket and three cages for each material are tested. Considering three different materials, a total of 810 force measurements are performed. A theoretical model is developed to predict the roller push-out forces in polymeric cages with different materials and pocket geometries. The model is developed by estimating the deformed region of the cage as a cantilever beam with a parabolic profile. An empirical coefficient is reposed in the model to compensate for the assumptions applied to the model. Experimental results showed that a fixed coefficient gives accurate results for all the geometries and materials, which confirms the validity of the approach adopted in this paper for modeling such problems. Considering the geometrical and material tolerances, force limits predicted by the model cover all the forces measured for a specific pocket with excellent accuracy and consistency.

## Introduction

Roller bearings are prominent components in rotating machinery, and their cost and performance directly affect the entire system. Typical roller bearings comprise an outer ring, an inner ring, balls or rollers as rolling elements, and a cage. The cage (also known as a bearing retainer or roller separator) is a component in roller bearings, and the primary functions of the cage are:

- separating the rolling elements to reduce friction in the bearing
- optimizing the load distribution by evenly spacing the rolling elements
- guiding the rolling elements
- keeping the rolling elements of separable bearings in position when a bearing ring is removed for mounting or dismounting
- providing lubrication by a solid film (coating or cage material) or functioning as an oil reservoir

All cages produce frictional forces that increase the starting and running torque of the bearings. Therefore, cages are designed in various configurations and materials to improve the performance of the bearings in different applications such as high speed, torque-sensitive, and extreme environment applications^1^.

There are four types of cages, namely: stamped metal, machined metal, pin-type, and polymeric cages. The polymeric cages, among others, produce less friction due to their good sliding properties and enable high speeds. These cages lower the risk of seizure and secondary damage in poor lubrication conditions because they can survive with a low amount of lubrication for some time. Furthermore, polymeric cages can be complex in shape while being inexpensive. These cages are usually made of polyamide 46 (PA46), polyamide 66 (PA66), polyphthalamide (PPA), polyether ether ketone (PEEK), polyethylene (PE), and polytetrafluoroethylene (PTFE). These cages are usually made in very large quantities, so the costs of molds and process automation are very high. Therefore, various research studies have focused on prototype samples to understand or verify the effects of manufacturing processes, geometrical dimensions, and material properties on different functions of the cages. For instance, Gaydamaka^2^ determined the relationships between structural elements and their functions in roller bearings and studied the effects of increasing the number of rollers in the cage. Williams^3^ investigated a new retainer for cylindrical roller bearings made of fiberglass reinforced nylon. The cage characteristics set up as design goals in this study are the applicability of one retainer for various bearings, maximizing the number of rollers, being race or roller guided, minimizing the roller/cage drop, and low cost. Ghaisas et al.^4^ developed a six Degrees of Freedom (DOF) model to study cage instabilities in roller bearings considering the roller-race and roller-cage pocket clearances for light-load and high-speed conditions. The results show that the cage exhibits stable motion for small values of roller-race and roller-cage pocket clearances. Kohar et al.^5^ considered large, tapered roller bearing with flexible body cages to provide information about kinematic and dynamic relationships of steel and plastic cages under various operating conditions. Cui et al.^6^ presented the nonlinear dynamic differential equations of high-speed roller bearing considering the impact of roller dynamic unbalance. Ripanu et al.^7^ considered different manufacturing parameters on the precision of the windows main dimensions that exist in the bearing cages in the case of the double row tapered roller bearings. Empirical mathematical models able to offer information concerning the impact of manufacturing parameters on three main dimensions of the bearing cage windows were determined in this research. Cui et al.^8^ presented a method for measuring the imbalance in a small-sized cylindrical roller and considered the impact of the roller imbalance on the dynamic characteristics of a cage. Moreover, there are some studies on the static^9^ and dynamic^10^ numerical analysis of fiber-reinforced polymeric materials. The study presented here, focusing on the effects of material properties and geometrical parameters on the roller push-out forces in polymeric cages, addresses the gaps in current literature. This exploration is essential because the amount of push-out force should be kept in a certain range. This range is defined by compromising between the highest allowable force required to eject the mold inserts and the lowest acceptable force needed to keep the rollers in position. To answer this question, a polymeric cage with 18 different pocket geometries is designed to investigate the effect of four main geometrical parameters. An innovative tooling system is designed and fabricated, enabling low-volume production of polymeric cages. Next, cages with different materials (PA46, PA66, PPA) are manufactured through injection molding, and the push-out forces are measured through a set of measurements. Finally, a mathematical model considering different cage geometries and materials is developed, and the results are validated by the experiments.

## Design and manufacturing of cages

**Figure 1:**
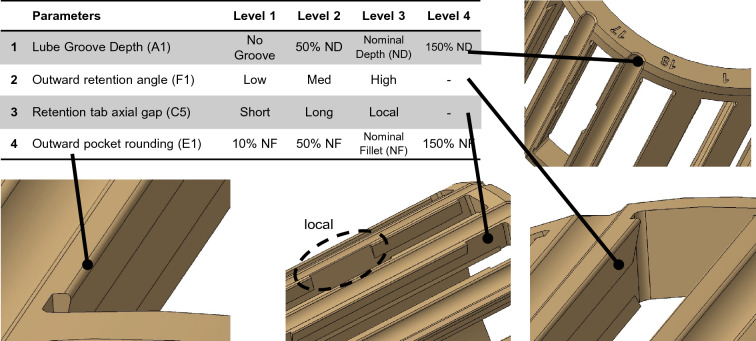
Selected geometrical parameters, their variation levels, and graphical representation.

To experimentally investigate the effect of the selected materials and geometrical parameters on the push-out force variation and later validate the theoretical model, a special polymeric cage was designed in this work. Another purpose of this design was to explore how the selected design parameters and their interactions can affect the production of the polymeric cages, i.e., the injection molding process and the required tooling system. Among all the possible design parameters, four geometrical parameters were selected based on their influence on the push-out force and considering mold design limitations. The selected design parameters, their variation levels, and the graphical representation of each parameter are indicated in Fig. 1.

Lube grooves are semi-circular grooves considered on the outer surface of the cage mainly for allowing additional lubrication into the bearing. The existence of this groove and its depth (A1) can affect the push-out force by allowing the retention tab to deform while the roller is moving outward. In a similar mechanism, another function of this feature is to assist mold inserts being removed from the pockets during the molding process—this action is here called mold insert extraction. The angle of the retention tab (F1) is another important geometrical parameter that directly affects the push-out force. This angle differentiates the retention tab from the rest of the pocket and creates the undercut feature that limits the mold insert extraction. Parts of the retention tab are usually cut off to reduce the undercut area, thus easing the mold insert extraction. The length and location of this cut-off area (C5) also impacts the push-out force in the same manner. The radius of the fillet on the outward edge of the retention tab, which is here called outward pocket rounding (E1), is another important geometrical parameter that influences both the insertion and extraction of the rollers in and out of the pocket. The larger fillets also reduce the severity of the undercut feature and prevent the retention tab edge from being distorted during the molding process. Based on the variation levels considered for these geometrical parameters, a special polymeric cage was designed with 18 different pockets, as listed in Table 1.

**Table 1:**
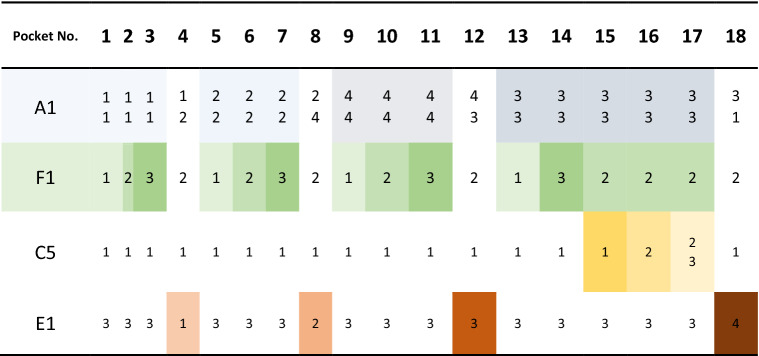
Configuration of the 18 pockets based on the selected geometrical parameters and their variation levels.

Color coded based on different parameters with darker shades denoting higher levels of each parameter.

**Figure 2:**
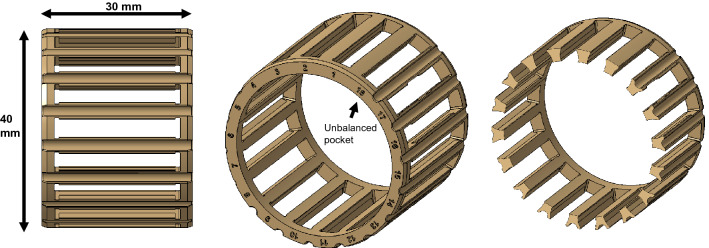
The design of the polymeric cage with 18 different pockets.

The designed polymeric cage and its overall dimensions are shown in Fig. 2 from several points of view. A pocket number was marked on the side of the cage to clearly distinguish each pocket. This numbering was also useful during experimentally measuring push-out forces to make sure proper values are reported for each pocket. As shown in Fig. 2, pocket 18 has no lube groove on one side and a deep lube groove on the other side. This is not a usual case in actual design of polymeric cages and causes unbalanced conditions in this pocket. Also, minor imbalance occurs in pockets 4, 8, and 12 due to the need for change in lube groove designs. Hence, pockets 4, 8, 12, and 18 were later excluded from the push-out force investigation to prevent erroneous feeding data into the modeling process.

**Figure 3:**
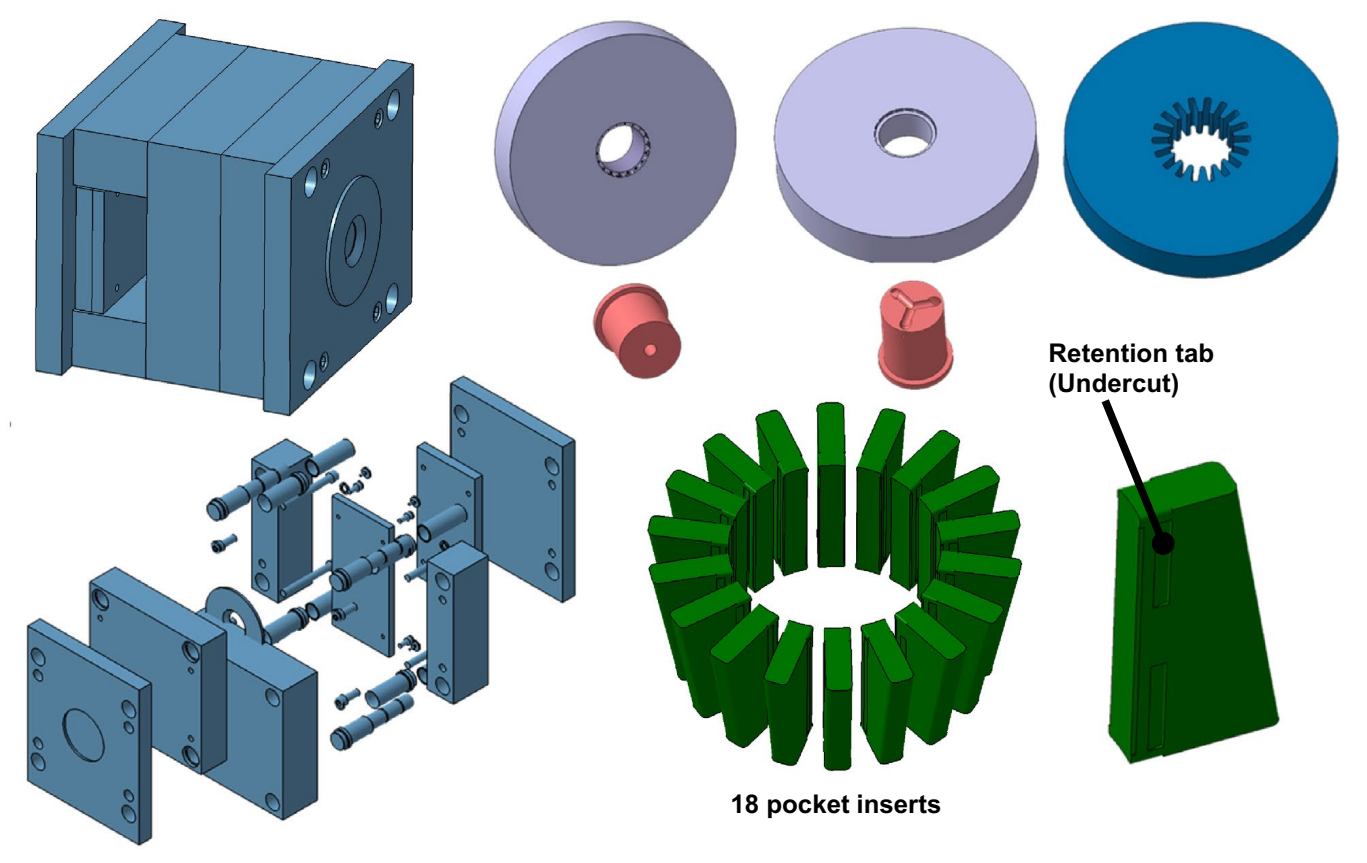
The specialized injection mold and its main parts (the models were not presented on the same scale).

An injection molding tool was specially designed and fabricated to manufacture the designed cage. This specialized tool and its main parts are demonstrated in Fig. 3. A modular approach was employed in this design of the tool by considering several inserts that can be modified and replaced to perform experimental iterations and develop new polymeric cages (beyond the scope of this work). Moreover, to reduce the complexity in this prototyping tool and cost-efficiently fabricate it, the mold was designed without considering any side actuator to extract the pocket inserts during the molding process. Hence, the injected part is ejected out of the cavity together with the mold inserts inside the pockets (see pocket inserts in Fig. 3). The pocket inserts were later manually removed from the cage, as discussed below.

After the mold was fabricated and tested, three series of polymeric cages were injection molded using the selected materials (PA46, PA66, and PPA). Prior to molding, pellets of the three selected materials were dried in an oven at 80 or 120 °C for 2–4 h to remove any residual moisture. The polymeric cages were then manufactured using an Engel Victory 30 injection molding machine. A minimum of ten cages were manufactured for each of the three selected materials with relevant molding parameters listed in Table 2.

**Table 2:** Experimental molding conditions for manufacturing polymeric cages of the three selected material systems.

Parameter	Unit	PA 46	PPA	PA 66
Drying temperature	°C	80	120	80
Drying time	h	2	4	2
Back pressure	bar	10	10	80
Melt temperatures	°C	315|315|310|310|295	325|325|320|320|315	300|300|295|285|285
Injection pressure	bar	150	150	150
Injection speed	mm/s	40.7	295	100
Holding pressure	bar	2000	1900	1400
Holding time	s	6	2.5	7
Cooling time	s	31.5	31.5	31.5
Screw rotation speed	m/s	0.28	0.28	0.22
Shot volume	cm^3^	7.44	7.44	7.44
Cycle time	s	40	35	41

**Figure 4:**
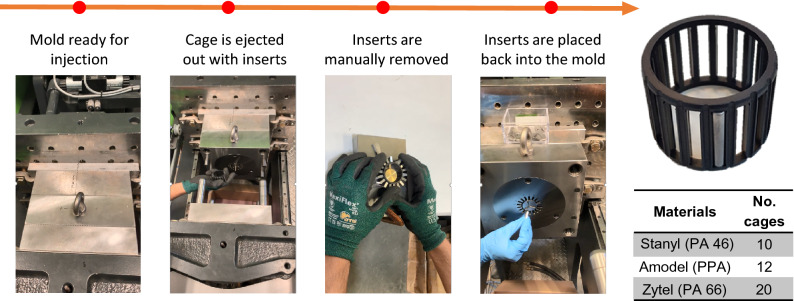
The procedure of manufacturing the polymeric cages and the final number of the molded parts for each material.

The overall procedure of manufacturing and assembling these cages is shown in Fig. 4, along with the final number of molded parts for each material. In the first step, the mold is in the closed position and ready for injection. After all the sequences of the injection molding are completed, the injected and solidified part (the cage) is ejected out of the cavity while the pocket inserts are still in the pockets. In the next step, the pocket inserts are removed from the cage using a manual tool designed for this purpose. Finally, all the pocket inserts are placed back inside their specific slots by matching their numbers. Now, the mold is ready for the next cycle and following the same procedure. The overall time of producing each cage was about 7 ± 1 mi, which is reasonable for prototyping the polymeric cages for research and development.

## Push-out force measurements

**Figure 5:**
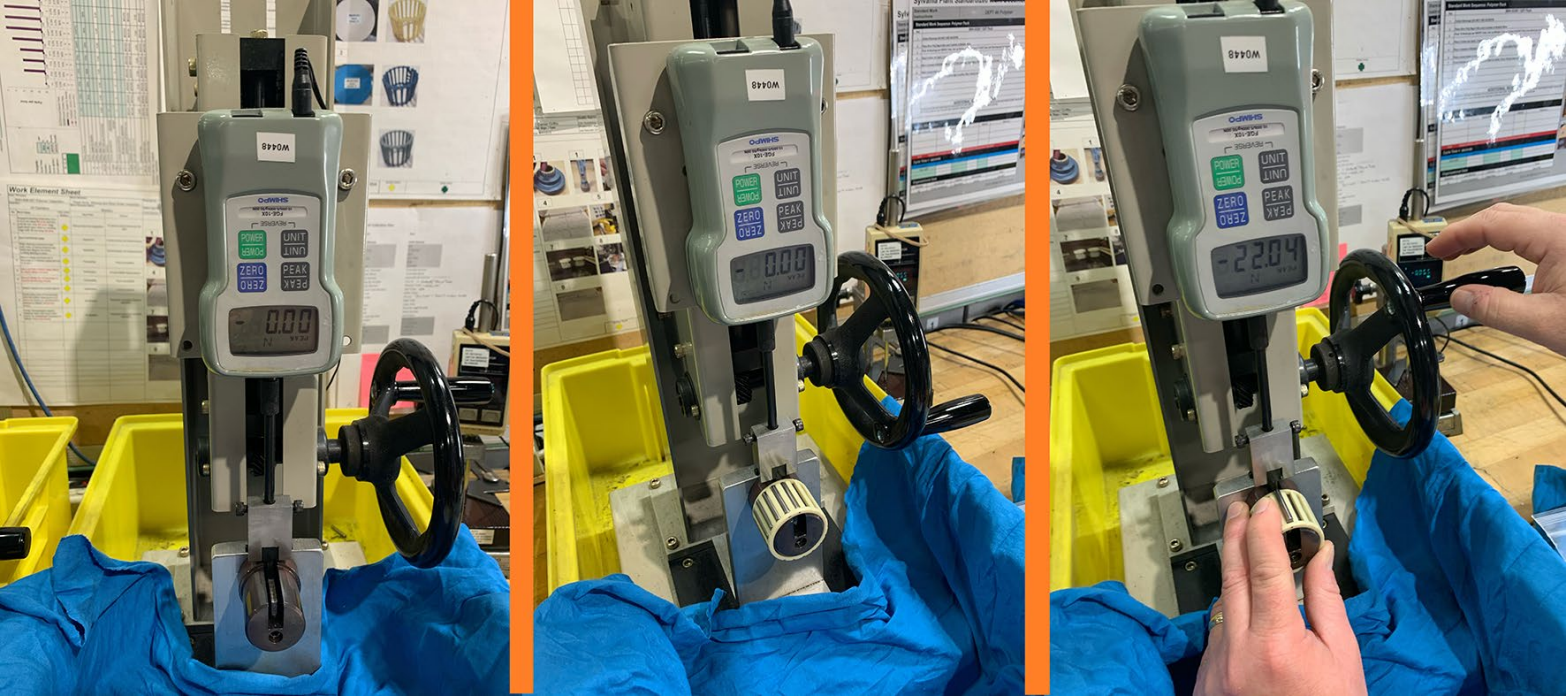
Experimental setup of the push-out force measurement.

The test setup is illustrated in Fig. 5. A Shimpo (FGE-10X) with a capacity of 50 N, resolution of 0.01 N, and accuracy of ± 0.2% is used to measure the push-out forces. This gauge can display real-time and peak forces. In this study, the peak value is recorded as a measure of the push-out force. A special component is designed and attached to the gauge hook. This component applies uniform pressure on the roller radially from inside the cage towards out of it without any contact with the cage. The force is applied to the roller gradually until the roller pops out, and the peak force is recorded.

The cages in this study are made of three different materials, and the same experiments are repeated for each material. The forces are measured five times for each pocket to find the push-out force variations in a specific cage. Moreover, three different samples for each material are tested to determine the manufacturing variations. The force measurements are performed on 18 pockets, out of which 14 completely symmetric pockets are reported here. Therefore, 210 measurements for each material and a total of 630 force measurements are performed on nine cages with different materials (three cages for each material). The results of the push-out force measurements are shown in Tables 3, 4 and 5.

**Table 3:** Measured forces of PA46 cages.

Pocket	1	2	3	5	6	7	9	10	11	13	14	15	16	17
**Sample 1—PA46**														
Attempt 1	20.56	37.38	36.5	22.58	41.56	40.07	13.3	23.8	29.43	15.29	32.82	28.3	26.41	33.6
Attempt 2	20.58	37.71	35.48	21.35	38.97	40.01	12.74	23	28.35	14.86	34.32	28.99	26.31	31.53
Attempt 3	20.1	40.69	36.01	21.92	39.28	38.72	12.12	23.25	26.9	14.65	33.49	27.33	25.94	32.87
Attempt 4	20.1	39.92	35.37	21.47	39.12	38.95	12.73	22.86	27.55	15.17	31.58	27.76	27.17	33.01
Attempt 5	23.57	36.88	35.5	21.75	40.98	38.24	12.82	23.4	27.29	14.61	32.77	28.51	26.6	33.15
**Sample 2—PA46**														
Attempt 1	27.45	38.16	44.75	26.35	40.03	44.38	13.77	22.5	26.86	16.94	35.19	28.55	25.02	33.21
Attempt 2	28.01	40.12	42.81	24.45	40.27	43.23	14.01	22.67	26.64	16.24	35.38	29.8	24.78	31.92
Attempt 3	27.41	39.74	41.45	27.2	38.98	45.21	14.09	23.12	26.8	15.39	33.66	29.43	25.8	31.98
Attempt 4	27.96	38.63	42.52	24.22	39.31	44.15	14.06	24.54	26.77	15.8	33.11	27.5	24.81	32.41
Attempt 5	28.7	37.95	42.18	24.01	38.99	42.57	13.77	24.8	27.26	15.66	35.4	30.29	25.42	31.91
**Sample 3—PA46**														
Attempt 1	27.08	35.54	42.71	22.24	36.35	41.48	12.47	21.17	28.4	15.03	34.15	28.02	23.78	29.94
Attempt 2	25.21	33.89	39.93	22.71	36.9	43.31	12.94	21.77	27.6	15.87	32.2	28.34	24.16	29.6
Attempt 3	24.23	33.93	39.32	22.44	36.84	41.59	13.03	21.7	28.66	15.96	31.41	27.68	24.25	29.91
Attempt 4	24.23	33.63	38.99	22.72	36.63	42.2	11.94	21.39	27.55	15.79	31.91	27.31	24.18	29.09
Attempt 5	25.16	34.42	40.24	22.97	36.26	45.45	12.39	20.72	27.94	16.39	31.48	28.37	24.41	30.05
**Average**	**24.69**	**37.24**	**39.58**	**23.23**	**38.7**	**41.97**	**13.07**	**22.71**	**27.6**	**15.58**	**33.26**	**28.41**	**25.27**	**31.61**
**Max force**	**28.7**	**40.69**	**44.75**	**27.2**	**41.56**	**45.45**	**14.09**	**24.8**	**29.43**	**16.94**	**35.4**	**30.29**	**27.17**	**33.6**
**Min force**	**20.1**	**33.63**	**35.37**	**21.35**	**36.26**	**38.24**	**11.94**	**20.72**	**26.64**	**14.61**	**31.41**	**27.31**	**23.78**	**29.09**

The [Bold] values are used in the subsequent calculations and plots.

**Table 4:** Measured forces of PA66 cages.

Pocket	1	2	3	5	6	7	9	10	11	13	14	15	16	17
**Sample 1—PA66**														
Attempt 1	24.34	38.3	42.64	19.52	36.55	40.83	12.16	21.08	24.39	12.75	32.56	27.43	21.82	28.85
Attempt 2	22.15	37.95	42.47	19.36	33.76	37.8	12.09	19.89	22.72	11.7	30.29	26.85	20.39	27.16
Attempt 3	21	36.86	41.39	18.51	34.25	37.09	12.63	20.09	22.87	12.21	30.47	26.23	20.83	27.11
Attempt 4	22.08	38.12	40.67	19.3	34.03	36.87	12.83	19.76	22.91	13.33	30.42	26.6	21.04	27.56
Attempt 5	21.16	36.88	40.16	19.31	33.65	35.72	12.3	19.68	21.38	12.07	28.07	24.82	19.97	28
**Sample 2—PA66**														
Attempt 1	24.14	38.95	43.84	19.39	37.59	42.97	14.13	22.28	23.94	13.6	32.91	26.63	22.39	29.78
Attempt 2	23.25	35.72	42.43	19.22	36.74	39.6	12.41	19.96	24.17	13.33	31.36	26.45	22.69	29.46
Attempt 3	23.57	36.63	42.27	18.23	35.07	38.73	12.21	21.3	24.03	13.17	30.94	25.8	21.65	29.43
Attempt 4	23.37	35.09	43.52	18.44	36.71	39.54	12.34	20.09	23.06	13.43	30.44	26.42	21.86	28.65
Attempt 5	22.37	35.47	42.87	19.1	35.64	38.62	11.98	20.08	23.42	13.01	29.07	25.99	21.94	27.74
**Sample 3—PA66**														
Attempt 1	24.54	41.63	44.98	20.07	39.99	41.61	12.67	20.68	24.22	16.04	31.94	29.65	25.18	31.57
Attempt 2	24.26	39.45	44.62	19.42	38.51	38.77	12.47	20.25	25.16	15.56	30.54	27.77	23.67	29.57
Attempt 3	23.88	38.84	45.42	19.81	37.66	37.91	12.46	19.53	24.26	14.92	29.77	26.82	23.41	30.32
Attempt 4	23.2	38.27	44.21	19.12	36.38	38.53	11.96	19.55	23.86	13.93	28.4	25.87	23.05	28.01
Attempt 5	22.47	38.45	42.3	19.16	35.88	37.14	11.95	20.09	23.91	14.43	28.21	26.25	23.19	28.13
**Average**	**23.05**	**37.77**	**42.92**	**19.20**	**36.16**	**38.78**	**12.44**	**20.29**	**23.62**	**13.57**	**30.36**	**26.64**	**22.21**	**28.76**
**Max force**	**24.54**	**41.63**	**45.42**	**20.07**	**39.99**	**42.97**	**14.13**	**22.28**	**25.16**	**16.04**	**32.91**	**29.65**	**25.18**	**31.57**
**Min force**	**21**	**35.09**	**40.16**	**18.23**	**33.65**	**35.72**	**11.95**	**19.53**	**21.38**	**11.7**	**28.07**	**24.82**	**19.97**	**27.11**

The [Bold] values are used in the subsequent calculations and plots.

**Table 5:** Measured forces of PPA cages.

Pocket	1	2	3	5	6	7	9	10	11	13	14	15	16	17
**Sample 1—PPA**														
Attempt 1	23.05	38.17	49.52	22.06	40.16	43.17	13.06	24.14	27.3	17.26	35.78	29.42	25.46	31.86
Attempt 2	22.04	35.9	46.4	22.33	37.56	39.08	14.63	21.69	25.76	17.53	35.46	28.78	24.57	30.12
Attempt 3	22.43	36.47	46.63	20.76	36.55	39.14	13.03	22.96	25.73	16.29	33.53	27.36	25.22	29.84
Attempt 4	22.79	36.86	45.81	22.06	39.01	41.14	13.42	23.13	26.37	16.87	33.8	28.69	24.81	30.42
Attempt 5	23.49	36.58	45.91	21.93	37.88	39.23	15.05	22.26	25.81	16.33	32.42	27.46	24.37	29.67
**Sample 2—PPA**														
Attempt 1	23.05	40.91	48.96	21.33	40.86	41.96	13.63	23.01	25.99	16.13	34.17	27.01	24.84	30.79
Attempt 2	22.45	40.22	45.29	21.88	37.02	42.11	12.52	22.2	25.14	15.7	31.97	27.43	25	31.1
Attempt 3	23.71	37.24	43.05	20.58	35.5	37.4	12.84	22.71	24.62	16.37	33.27	26.56	24.12	31.05
Attempt 4	22.33	38.83	42.41	19.28	35.18	36.65	12.58	21.72	24.44	14.97	32.28	25.99	23.56	30.92
Attempt 5	23.55	37.37	41.66	20.35	33.92	37.35	12.86	21.94	24	15.03	32.4	26.62	23.83	29.37
**Sample 3—PPA**														
Attempt 1	22.54	39.28	47.75	20.57	39.17	40.82	13.11	22.01	25.29	15.17	34.2	26.83	24.24	31.03
Attempt 2	22.57	36.11	44.72	20.18	37.05	38.74	12.74	21.76	24.69	14.99	31.21	25.85	24.19	30.86
Attempt 3	21.85	36.22	42.04	20.37	36.02	38.15	11.99	21.74	24.77	15.24	29.67	26.61	23.94	29.38
Attempt 4	21.66	36.18	42.62	21.25	36	38.78	11.93	21.5	23.92	15.89	31.16	25.88	23.88	30.36
Attempt 5	21.03	37.07	43.33	20.18	36.59	36.39	11.95	22.07	23.94	14.7	31.03	26.59	25.82	29.59
**Average**	**22.57**	**37.56**	**45.07**	**21.01**	**37.23**	**39.34**	**13.02**	**22.32**	**25.18**	**15.9**	**32.82**	**27.14**	**24.52**	**30.42**
**Max force**	**23.71**	**40.91**	**49.52**	**22.33**	**40.86**	**43.17**	**15.05**	**24.14**	**27.3**	**17.53**	**35.78**	**29.42**	**25.82**	**31.86**
**Min force**	**21.03**	**35.9**	**41.66**	**19.28**	**33.92**	**36.39**	**11.93**	**21.5**	**23.92**	**14.7**	**29.67**	**25.85**	**23.56**	**29.37**

The [Bold] values are used in the subsequent calculations and plots.

## Push-out force modeling

A predictive model is needed to calculate the push-out force in the roller bearings and characterize the effect of each design parameter on the push-out force. Being analytical, this model explicitly reveals the effect of each design parameter on the push-out force, which will be helpful in design optimization. Furthermore, the critical dimensions and their manufacturing tolerances can be introduced based on the constraints on the push-out forces.

**Figure 6:**
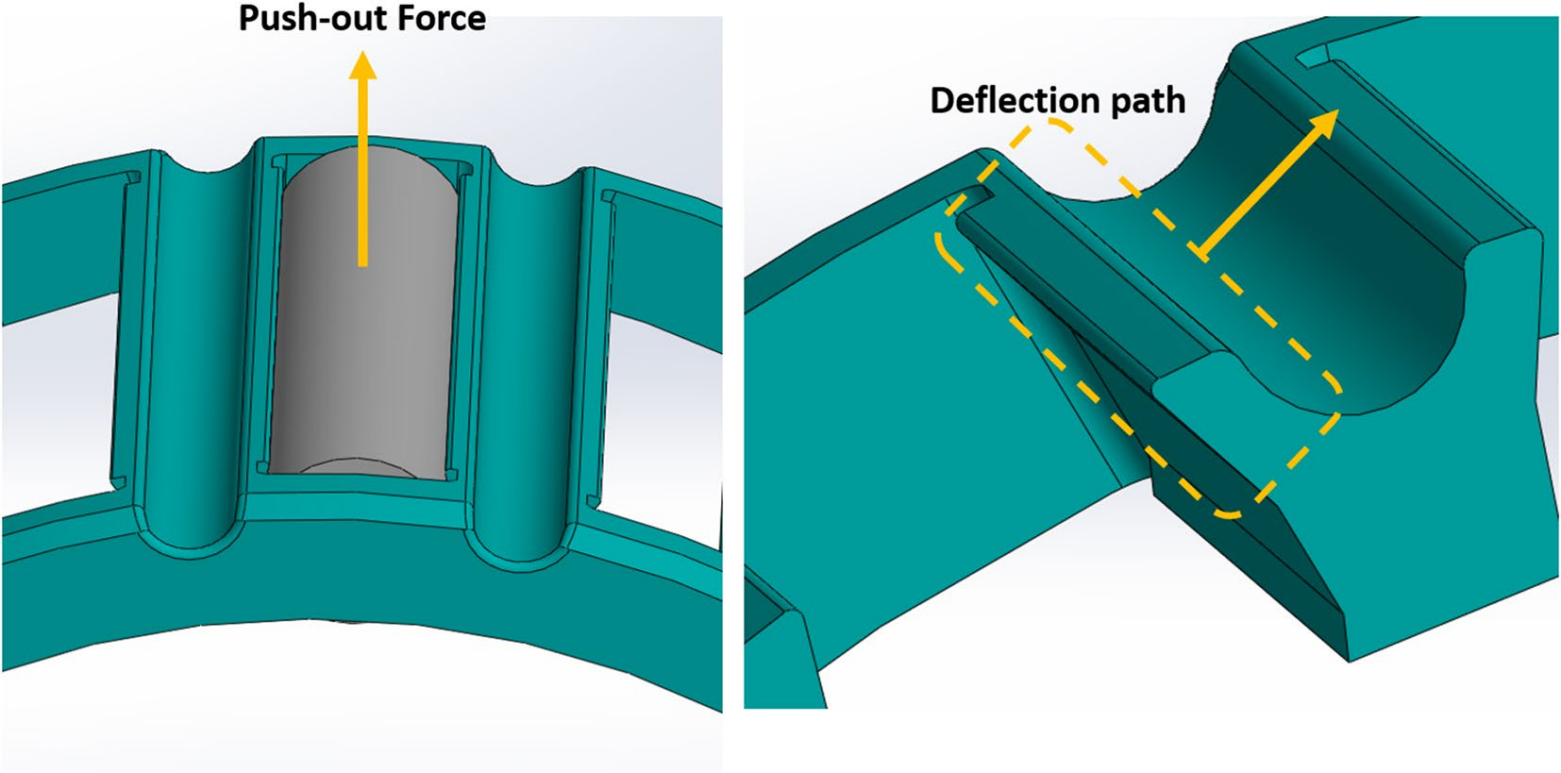
Push-out force and deflection path directions.

**Figure 7:**
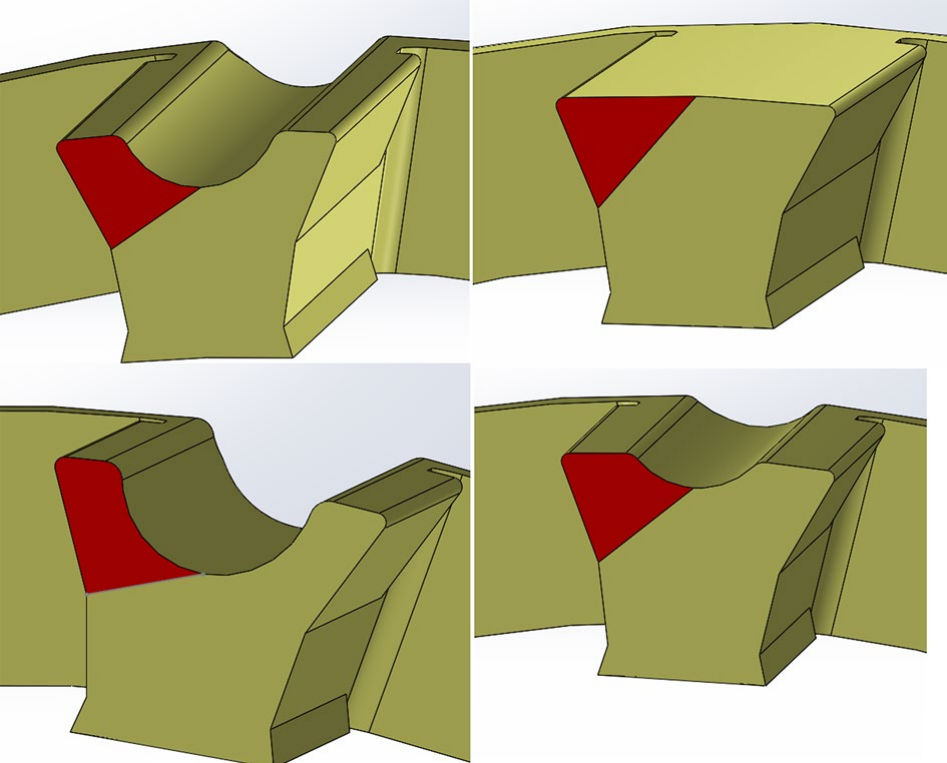
Region of significant deformation.

The first step in modeling this complex problem is to simplify and estimate the problem. Since the push-out force is uniformly applied to the roller and there is no change in parameters along the roller axis, the modeling is carried out based on the cage profile in the plane normal to the roller axis. By considering the deformation mechanism of the cages when the roller is being pushed out, it is realized that when the force is applied to the roller, the cage retention is deformed, and the roller comes out (Fig. 6). This deformation is confined to some region near the retention tip, as shown in Fig. 7.

Since the roller and the cage surfaces are very smooth, and there is not much friction between them, the frictional forces are neglected. The part of the material that undergoes significant deformation while the roller is pushed out is considered as a cantilever beam to model the cage deformation and relate it to the force required to push out the roller. The Region of Significant Deformation (ROSD) is considered as the region above the line connecting the bottom of the lube groove to the bottom of the retention, as shown in Fig. 7.

**Figure 8:**
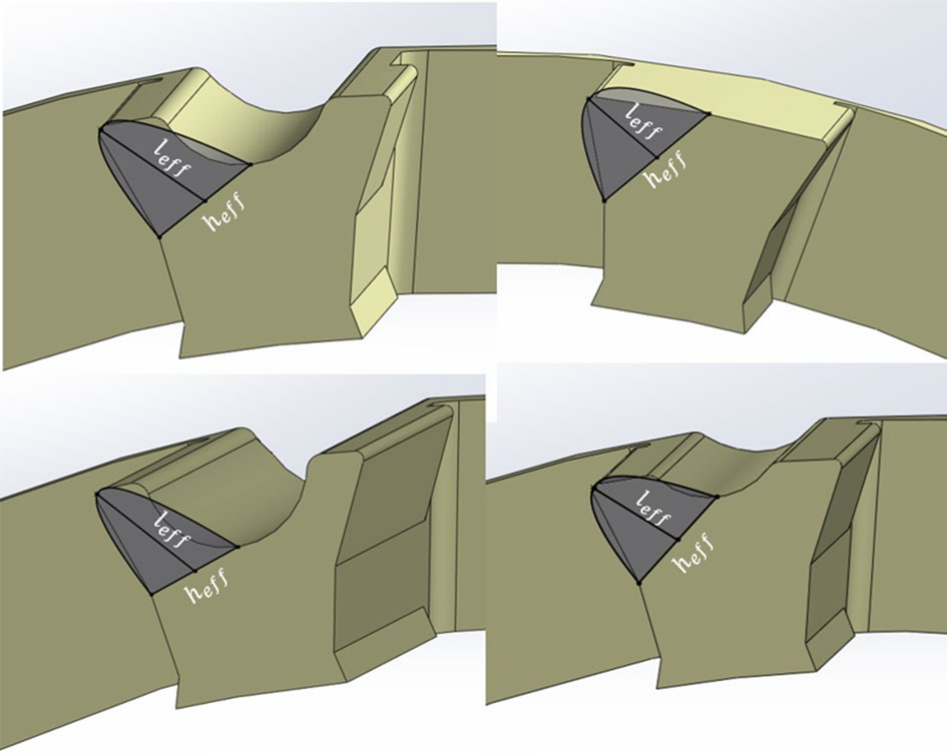
Modeling the deformed areas of the cages as a parabolic curve.

To model the ROSD as a cantilever beam, a reference height and length are needed. The ROSD height is termed as effective height (the length of the line connecting the bottom of the lube groove to the bottom of the retention). The ROSD length is termed as effective length (the length of the line connecting the middle of the effective height line to the tip of the retention), as shown in Fig. 8. The cage geometry is not regular and changes from cage to cage. Therefore, a profile that adapts itself to the cage geometry and provides an accurate estimation is needed. Here, a parabolic profile with the effective height as its height and the effective length as its length is introduced to estimate the ROSD and capture various cage profiles (see Fig. 8).

**Figure 9:**
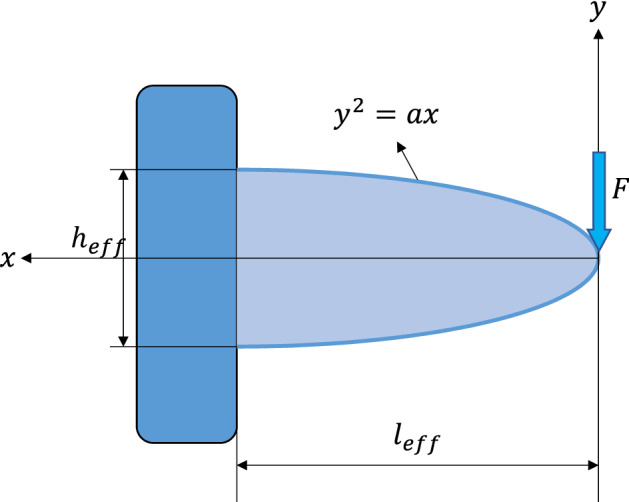
Cantilever beam with a parabolic profile.

Therefore, the problem is considered as a cantilever beam with a parabolic profile and a concentrated load at the tip, as depicted in Fig. 9.

The constant $a$ in the equation ${y}^{2}=ax$ shown in Fig. 9 can be found by substituting effective length (${l}_{eff}$) for $x$, and half of the effective height (${h}_{eff}$) as $y$ and solving for the constant $a$, which gives $a=\frac{{h}_{eff}^{2}}{4{l}_{eff}}$.

Next, the equation for the deflection of the parabolic cantilever beam is derived based on the strain energy and Castigliano’s theorem. Since the width of the beam is large compared to other dimensions, the effects of shear forces are also considered. The strain energy of a beam due to bending is defined as:

1 $${U}_{b}={\int }_{0}^{{l}_{eff}}\frac{{M}^{2}}{2EI}dx$$

And, the strain energy due to shear for a rectangular cross-section is defined as:

2 $${U}_{s}={\int }_{0}^{{l}_{eff}}\frac{0.6{F}^{2}}{GA}dx$$

where $M$ is the moment, $F$ is the shear force, $E$ is Young’s modulus, $G$ is the shear modulus, and $I$ and $A$ are the second moment of area and the cross-sectional area, respectively, and are defined as follows:

3 $$I=\frac{{B}_{5}\times {\left(2y\right)}^{3}}{12}=\frac{{B}_{5}{\left({h}_{eff}\right)}^{3}}{12}{\left(\frac{x}{{l}_{eff}}\right)}^\frac{3}{2}$$

4 $$A={2B}_{5}y={{B}_{5}h}_{eff}\sqrt{\frac{x}{{l}_{eff}}}$$

where ${B}_{5}$ is the width of the beam. The total strain energy of the beam would be:

5 $$U={U}_{m}+{U}_{s}={\int }_{0}^{{l}_{eff}}\frac{12{F}^{2}{x}^{2}}{2E{B}_{5}{h}_{eff}^{3}}{\left(\frac{{l}_{eff}}{x}\right)}^\frac{3}{2}dx+{\int }_{0}^{{l}_{eff}}\frac{0.6{F}^{2}}{{B}_{5}{h}_{eff}G}\sqrt{\frac{{l}_{eff}}{x}}dx$$

Performing the integrations gives:

6 $$U={U}_{m}+{U}_{s}=\frac{4{F}^{2}{l}_{eff}^{3}}{E{B}_{5}{h}_{eff}^{3}}+\frac{1.2{F}^{2}{l}_{eff}}{G{B}_{5}{h}_{eff}}$$

Next, Castigliano’s theorem can be used to calculate the deflection of the beam. Castigliano’s theorem states: “the first partial derivative of the total strain energy in a structure with respect to the force applied at any point is equal to the deflection at the point of application of that force in the direction of its line of action”. Thus, the deflection of the beam can be calculated as follows:

7 $$\delta =\frac{\partial U}{\partial F}=\frac{8F{l}_{eff}^{3}}{E{B}_{5}{h}_{eff}^{3}}+\frac{2.4F{l}_{eff}}{G{B}_{5}{h}_{eff}}$$

Solving for the force $(F)$ and introducing a correction factor $(C)$ to compensate for the simplifications and estimations gives:

8 $$F=C\cdot \frac{\delta }{\frac{8}{E{B}_{5}}{\left(\frac{{l}_{eff}}{{h}_{eff}}\right)}^{3}+\frac{2.4{l}_{eff}}{G{B}_{5}{h}_{eff}}}$$

**Figure 10:**
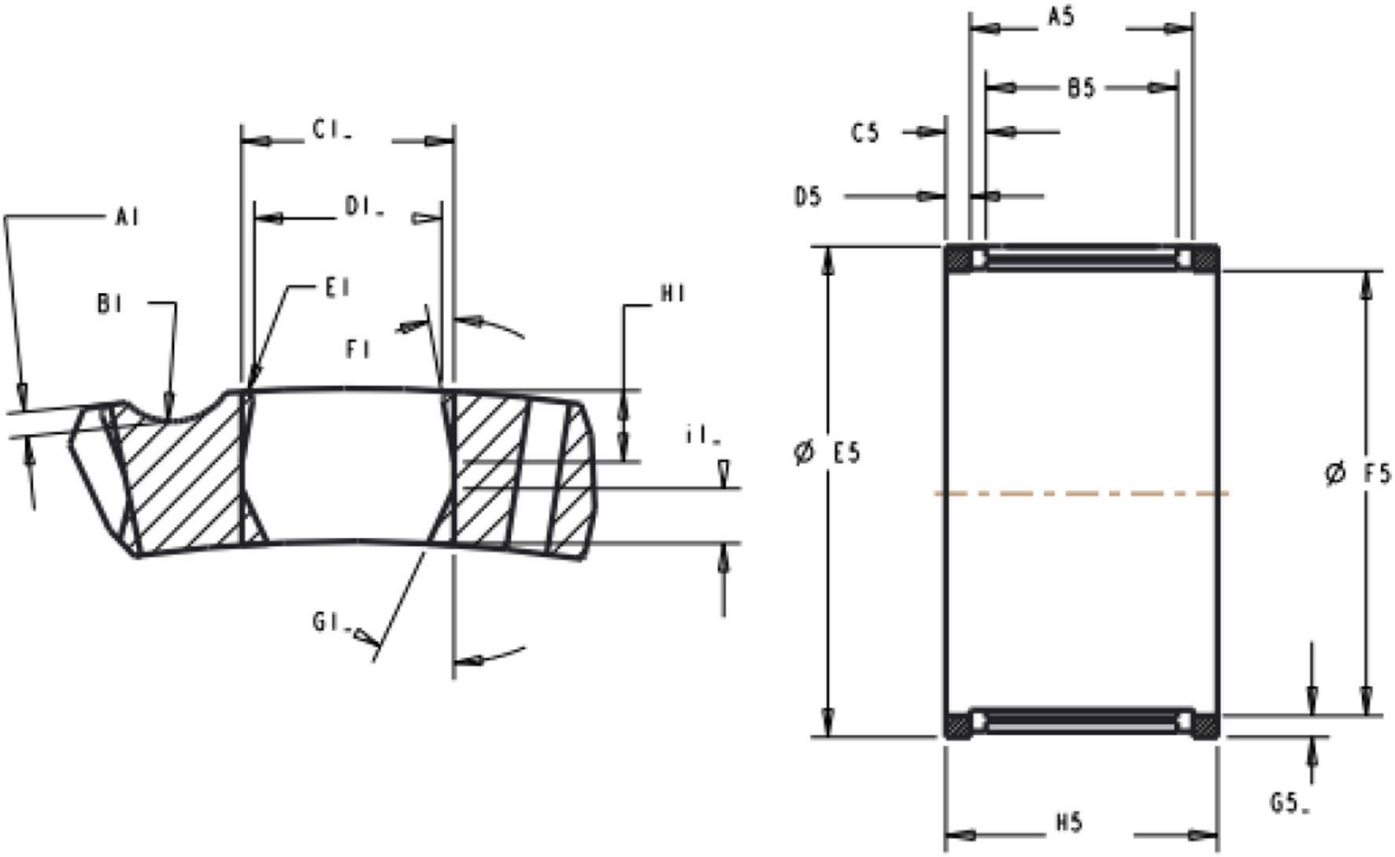
The important geometrical features and dimensions of polymeric cages notations.

The deflection $\delta$ here is the difference between the roller diameter and the outer pocket width (${D}_{1}$). The notations used here are shown in Fig. 10.

The names of the features in Fig. 10 are listed in Table 6.

**Table 6:** Names of the cage features.

Feature	Name
${A}_{1}$	Lube groove depth
${B}_{1}$	Lube groove radius
${C}_{1}$	Pocket width (center)
${D}_{1}$	Pocket width (inner)
${E}_{1}$	Outward pocket rounding
${F}_{1}$	Outward retention angle
${G}_{1}$	Inward retention angle
${H}_{1}$	Outward retention feature location
${i}_{1}$	Inward retention feature location
${A}_{5}$	Pocket width
${B}_{5}$	Retention width
${C}_{5}$	Retention tab axial location
${D}_{5}$	End rim width
${E}_{5}$	Outer diameter
${F}_{5}$	Inner diameter
${G}_{5}$	Rim cross-section (thickness)
${H}_{5}$	Cage width
${C}_{6}$	Roller diameter

**Figure 11:**
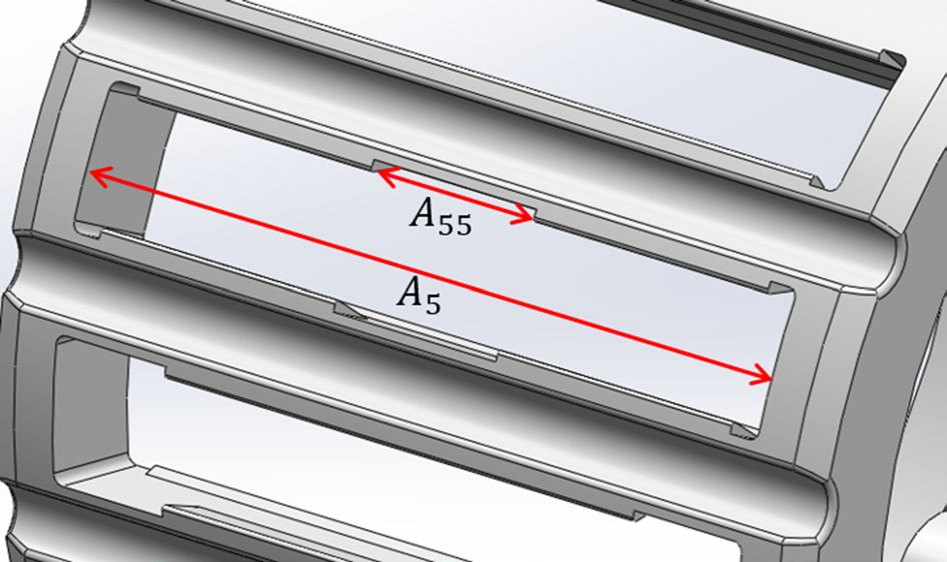
Pocket width and retention discontinuity.

Furthermore, some of the cage pockets have a discontinuity (${A}_{55}$ in Fig. 11) in their retention that must be considered in the modeling.

Now, some extra terms are needed to account for the end rim width (${D}_{5}$) and retention discontinuity (${A}_{55}$). Also, since the fraction $\frac{{l}_{eff}}{{h}_{eff}}$ is sometimes less than one, and other times very close to one, and the results showed more stability with the 0.5 power for the ratio of the effective length over effective height, the power three is replaced by 0.5 in Eq. (8), and the final push-out force model is cast as follows:

9 $$F=C\cdot \frac{\left({C}_{6}-{D}_{1}\right)\left(1+\frac{{A}_{55}}{{A}_{5}}\right)\left(1+\frac{{A}_{5}-{B}_{5}}{{A}_{5}}\right)}{\frac{8}{E{B}_{5}}{\left(\frac{{l}_{eff}}{{h}_{eff}}\right)}^{0.5}+\frac{2.4{l}_{eff}}{G{B}_{5}{h}_{eff}}}$$

where the effective height and length are shown in Fig. 8, and are defined as:

10 $${h}_{eff}=\sqrt{{\left(\frac{\left({E}_{5}-2{H}_{1}\right)\pi {C}_{2}}{720}-\frac{{C}_{1}}{2}\right)}^{2}+{\left({H}_{1}-{A}_{1}\right)}^{2}}$$

11 $${l}_{eff}=\sqrt{{\left(\frac{{h}_{eff}}{2}\right)}^{2}+{\left(\frac{{H}_{1}}{\mathrm{cos}({F}_{1})}\right)}^{2}-\frac{{h}_{eff}{H}_{1}}{\mathrm{cos}({F}_{1})}cos\left({cos}^{-1}\left(\frac{{H}_{1}-{A}_{1}}{{h}_{eff}}\right)+{F}_{1}\right)}$$

In Eqs. (9)–(11), $E$ and $G$ are material properties, and all other parameters are standard geometrical parameters that are readily available. The correction factor $C$ is determined by substituting the geometrical parameters and material properties in Eq. (9) and setting the magnitude of the push-out force $F$ to the mean of the experimental values. There are 14 different pocket geometries and three materials, which means there are 42 different cases. Interestingly the correction factors for all 42 cases were very close to each other that we could set one single number for $C,$ which works for all materials and geometries. This is a significant achievement proving the ability of the model to consider all the parameters and their effects correctly. The correction factor turned out to be $C=0.005,$ and the final model can be written as:

12 $$F=0.005\frac{\left({C}_{6}-{D}_{1}\right)\left(1+\frac{{A}_{55}}{{A}_{5}}\right)\left(1+\frac{{A}_{5}-{B}_{5}}{{A}_{5}}\right)}{\frac{8}{E{B}_{5}}{\left(\frac{{l}_{eff}}{{h}_{eff}}\right)}^{0.5}+\frac{2.4{l}_{eff}}{G{B}_{5}{h}_{eff}}}$$

## Results and discussion

**Figure 12:**
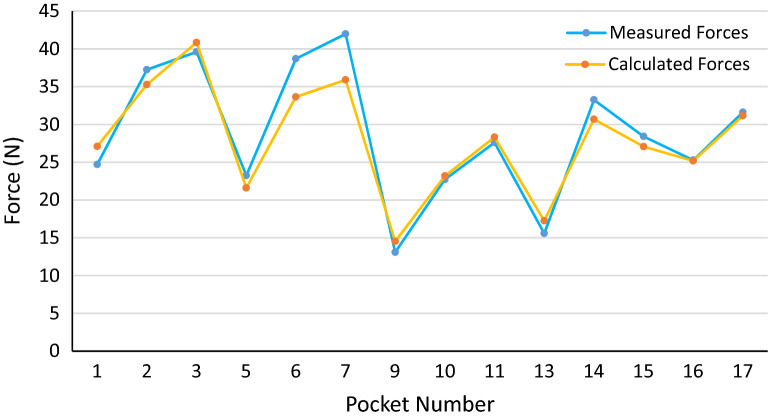
Measured and calculated push-out forces in PA46 cages.

**Figure 13:**
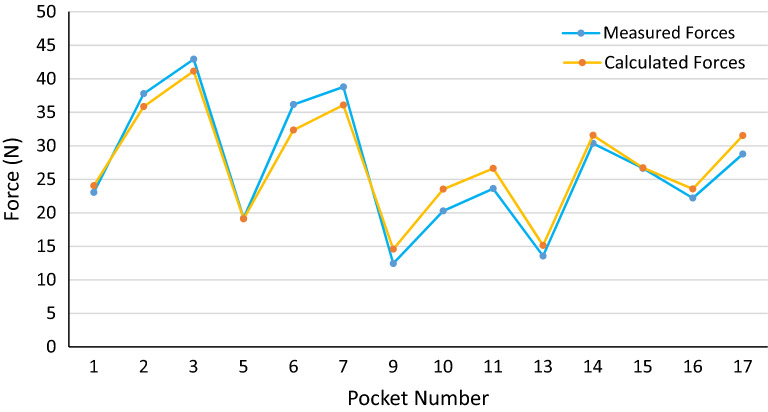
Measured and calculated push-out forces in PA66 cages.

**Figure 14:**
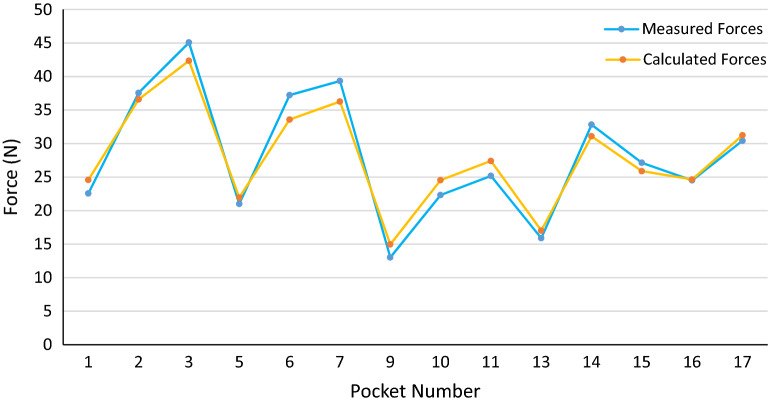
Measured and calculated push-out forces in PPA cages.

In this section, the results of the measurements and calculations based on the proposed model are presented. It is noteworthy that the most critical dimension affecting the push-out force is the pocket width (${D}_{1}$), so the tolerances of this dimension must be tight to keep the push-out forces in the allowable region. The shrinkage of the materials has a significant effect on the pocket width, so it should be considered very carefully. The pocket widths of the cages in this study vary slightly from material to material because one mold is used for all the materials, deviating from nominal dimensions. Hence, the actual pocket width is measured in different cages, and the average values are used for each material. The diagram of the measured forces (experimental) and calculated forces (theoretical) for different materials are depicted in Figs. 12, 13 and 14.

**Figure 15:**
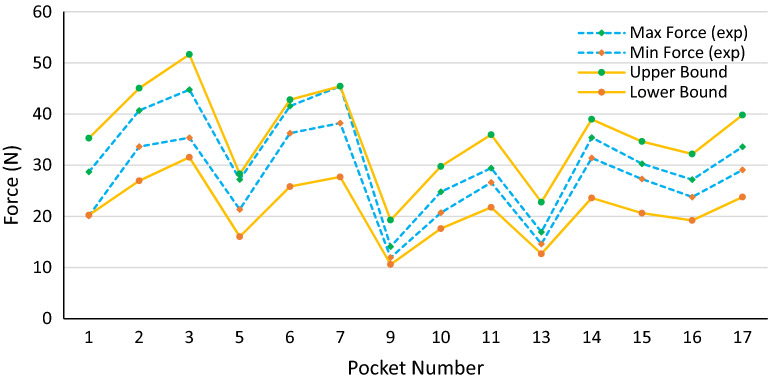
The range of measured push-out forces and calculated upper and lower bonds with six sigma variations in material properties, 0.02 mm in pocket width, and 0.05 mm in other dimensions for PA46 cages.

**Figure 16:**
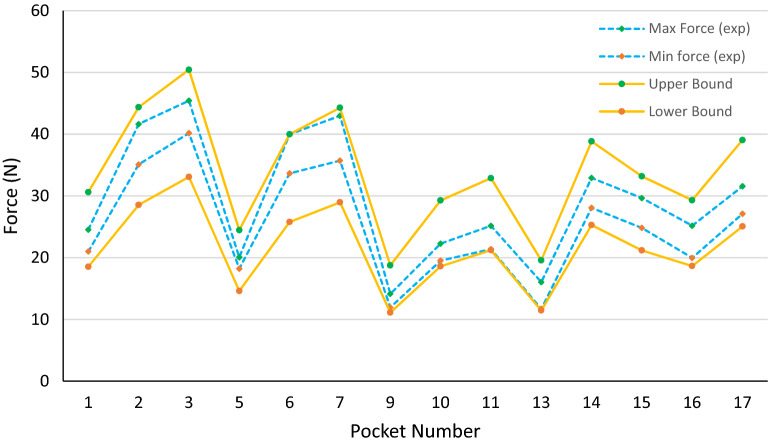
The range of measured push-out forces and calculated upper and lower bonds with six sigma variations in material properties, 0.02 mm in pocket width, and 0.05 mm in other dimensions for PA66 cages.

**Figure 17:**
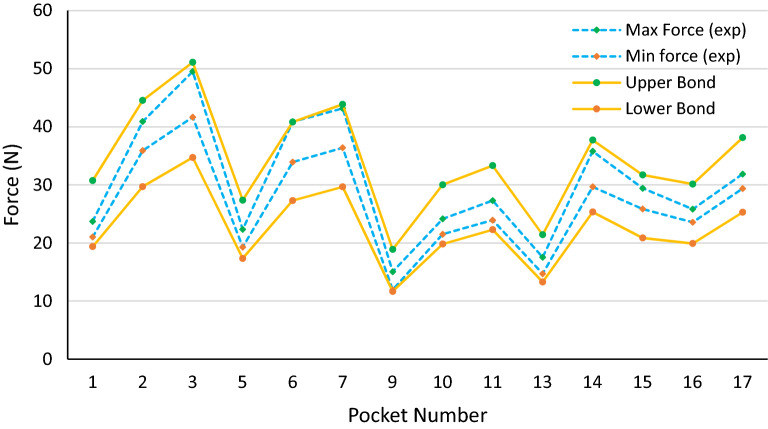
The range of measured push-out forces and calculated upper and lower bonds with six sigma variations in material properties, 0.02 mm in pocket width, and 0.05 mm in other dimensions for PPA cages.

The average values for material properties and pocket width are considered in all the plots. However, the effects of manufacturing variations on push-out forces must be investigated to define the tolerances of different parameters. Five different coupons for each material are made, and tensile testing is performed to measure the material properties. The standard deviation of the material properties is calculated, and six-sigma is considered as the range of the material properties. As the pocket width is the critical dimension, its tolerance is defined as 0.02 mm, and all other parameters in Eq. (12) are considered to have 0.05 mm tolerance. Based on the tolerances mentioned above, the upper and lower limits of calculated forces along with minimum and maximum measured push-out forces are plotted in Figs. 15, 16 and 17.

As can be seen from the plots, all the measured forces are in the range predicted by the model. Therefore, this model can predict the minimum and maximum push-out forces for a specific design with certain material properties and cage dimensions tolerances. This is very helpful in the design stage because the designers must compromise between the minimum push-out force (for the roller not to pop out when delivered to the customer) and the maximum push-out force (the amount of force required to eject the mold inserts without damaging the cage). Moreover, the manufacturing tolerances of the cages can be defined based on the given range of push-out forces.

The fluctuations of measured forces in different samples root in the variations in material properties and, more importantly, in the dimensions of the cages. PPA has the highest consistency in terms of material properties followed by PA66, and PA46 showed the highest deviation in the tensile tests. However, the higher shrinkage and dimensional consistency come together in which PA46 is at the top of the list, followed by PA66 and PPA. This might be because the higher shrinkage makes ejecting the mold inserts easier. The plots reveal that the choice of material does not affect the push-out forces as the dimensional parameters do. For instance, increasing the outward retention angle (F1), which results in smaller pocket width, substantially raises the push-out force. A deeper lube groove reduces the effect of retention angle on the push-out force. Also, a deeper lube groove decreases the stiffness of the cage in the retention tab region, which leads to a decrease in the push-out force. Increasing the retention tab axial gap (C5) reduces the push-out force, while adding a local gap at the middle of the retention tab increases this force.

## Conclusion

In this paper, a special polymeric cage is designed and manufactured through injection molding to study the effects of geometrical dimensions and material properties on the amount of push-out force. A specialized injection molding tool was designed and fabricated to realize manufacturing the cages made of three selected materials (PA46, PA66, PPA). The push-out forces were experimentally measured several times on different cages to determine manufacturing and measurement variations. An analytical model for predicting the push-out forces in roller bearings was presented. An empirical coefficient was introduced to the model, which is determined by the experimental push-out force measurements. The correction factor for all the geometries and materials were very close to each other such that one single number could be used as the correction factor in the model. This acknowledges the procedure adopted here to model such problems. Finally, the calculated forces were compared to measured forces, and a very good agreement was achieved. The proposed model is analytical, which shows the effects of each parameter on the amount of push-out force and helps engineers in the design stage where the dimensions of the cages are being determined, and size optimization is performed. Furthermore, the manufacturing tolerances can be defined using this model by introducing the push-out force limits.

The results of this study show that it is better to select the cage material based on the operational conditions of the roller bearing as the amount of push-out force can be adjusted by the dimensional parameters more effectively. The most effective dimension on the push-out force is the pocket width which is controlled by the outward retention angel. Being heavily dependent on the retention angle, the push-out force fluctuates with small variations in this parameter. A deep lube groove is an effective way to reduce the sensitivity of the push-out force to the retention angel and decrease the deviation of the measured forces. Therefore, it is advisable to maximize the lube groove depth and minimize the outward retention angle to achieve the best consistency in the push-out forces.
